# Two new species of *Dzhanokmenia* (Hymenoptera, Eulophidae) from China, with first report on a host association for the genus

**DOI:** 10.3897/zookeys.1009.57556

**Published:** 2021-01-04

**Authors:** Qin Li, Chao Wang, Hong-Ying Hu

**Affiliations:** 1 College of Life Science and Technology, Xinjiang University, 666 Shengli Road, Tianshan District, Urumqi, Xinjiang, 830046, China Xinjiang University Urumqi China; 2 Xinjiang Key Laboratory of Biological Resources and Genetic Engineering, 666 Shengli Road, Tianshan District, Urumqi, Xinjiang, 830046, China Xinjiang Key Laboratory of Biological Resources and Genetic Engineering Urumqi China

**Keywords:** Cecidomyiidae, Chalcidoidea, desert, taxonomy, Xinjiang

## Abstract

Two new species of *Dzhanokmenia* Kostjukov (Hymenoptera: Eulophidae: Tetrastichinae), *D. stefaniolae* Li, Wang & Hu, **sp. nov.** and *D. yuxuannis* Li, Wang & Hu, **sp. nov.**, are described and illustrated from Xinjiang Uyghur Autonomous Region, China. *D. stefaniolae* was reared from stem-galls made by *Stefaniola* sp. (Diptera: Cecidomyiidae) on black saxaul, *Haloxylon ammodendron* (Chenopodiaceae); *D. yuxuannis* was collected by sweeping from both black saxaul and white saxaul, *H. persicum*, in Beishawo Desert near Fukang. A key to females of all known species of *Dzhanokmenia* is provided.

## Introduction

Tetrastichinae is the largest subfamily of Eulophidae (Hymenoptera: Chalcidoidea). At present, this subfamily includes about 1900 species in more than 100 genera throughout the world (Noyes 2020).

Kostjukov (1977) regarded *Tetrastichus* (*Dzhanokmenia* Kostjukov), with type-species *Tetrastichus bibikovae* Dzhanokmen, as one of the 17 subgenera of the genus *Tetrastichus* Haliday (*sensu lato*). Graham (1991) revised the European Tetrastichinae, recognizing 28 valid genera including *Dzhanokmenia*, which he upgraded to genus level. The reasons why it was given a generic rank were discussed in Li et al. (2016). Kostjukov (2014) and Kostjukov and Kosheleva (2014, 2015) described three species of *Dzhanokmenia*. Li et al. (2016) described three more species of the genus during an expedition to study insect biodiversity of the Junggar Basin in northern Xinjiang Uyghur Autonomous Region (hereafter Xinjiang), China. Up to now, 13 valid species of *Dzhanokmenia* have been described from arid and semi-arid areas of southern Russia and Central Asia; however, their hosts remained unknown.

Here we describe two new species of *Dzhanokmenia* from rearings and collections, and provide a key to females of all known species of the genus.

## Materials and methods

### Parasitoid wasp collection and rearing

Our research group (Hong-Ying Hu, Qin Li, Wan Yin, Ya-Jie Zhu, Feng Li) collected many insects in 2015 by sweeping with a net and searching for galls on black saxaul, *Haloxylon ammodendron*, and white saxaul, *H. persicum* (Chenopodiaceae), in Beishawo Desert, near Fukang (44°22'29"N to 44°22'49"N, 87°52'57"E to 87°52'58"E, 401–446 m). These galls were reared by Qin Li in the laboratory at room temperature (20–32 °C) and 22–60% relative humidity, with natural and fluorescent lighting of approximately 13:11 L:D, at the College of Life Science and Technology, Xinjiang University, Urumqi. The emerged insects were preserved in 100% ethanol after dying naturally, without any food or water for 2–3 days usually.

### Taxonomy

All the specimens were examined under a Nikon SMZ 745T stereomicroscope. The images were taken with a Nikon DS-Fi3 connected to a Nikon SMZ 25 stereomicroscope. All images were stacked with NIS-Elements software and arranged in plates using Adobe Photoshop.

Description of each new species is based on its holotype, with variation of some key morphological features in the paratypes summarized separately. Morphology and terminology follows Gibson (1997) except for the metasoma. Gibson (1997) used petiole + gaster for the metasoma. In the case of *Dzhanokmenia*, the petiole is sessile and metasoma is equal to gaster, the metasomal terga I–VII are synonym with gastral terga I–VII. Abbreviations of morphological terms used are: C3, claval segment 3; CC, length of costal cell; CL, length of clava; CW, width of clava; EH, height of eye; EL, length of eye; F1–F3, funicle segments 1–3; FWL, length of fore wing; FWW, width of fore wing; GL, length of metasoma; GW, width of metasoma; HL, head length; HW, head width; HWL, length of hind wing; HWW, width of hind wing; MFL, length of metafemur; MFW, width of metafemur; ML, length of mesosoma; MLL, length of midlobe of mesoscutum; MLW, width of midlobe of mesoscutum; MSP, malar space; MV, length of marginal vein; MW, width of mesosoma; OOL, distance between eye and posterior ocellus; PMV, length of postmarginal vein; POL, distance between posterior ocelli; PSV, length of parastigma; SL, length of scape; SMV, length of submarginal vein of fore wing; STL, length of scutellum; STV, length of stigmal vein; STW, width of scutellum; SW, width of scape.

An acronym for the depository of parasitoids is: ICXU, Insect Collection of College of Life Science and Technology, Xinjiang University, Urumqi, Xinjiang, China. All the parasitoids were identified by the first and second authors. *Stefaniola* sp. was identified to genus by Ke-Long Jiao (Department of Plant Protection, College of Horticulture and Landscape, Tianjin Agricultural University, Tianjin, China), in whose collection some of the voucher specimens of this gall-maker are deposited; the remainder are in ICXU.

The key is an update of that in Li et al. (2016).

## Results

Two new species of *Dzhanokmenia* Kostjukov are described and illustrated here. All specimens of one of the new species of *Dzhanokmenia* were reared from stem-galls made by *Stefaniola* sp. (Diptera: Cecidomyiidae) on *H. ammodendron*, which is the first host record for the genus. Specimens of the other new species were collected by sweeping on both *Haloxylon ammodendron* and *H. persicum*.

### Systematics

#### Dzhanokmenia

Taxon classificationAnimaliaHymenopteraEulophidae

Kostjukov, 1977

9F86DA0D-70D2-5027-855C-AEAFBB05AC16

Tetrastichus (Dzhanokmenia) Kostjukov 1977: 189. Type-species: Tetrastichus bibikovae Dzhanokmen, by original designation. Subsequent references: Kostjukov 1978: 430–467 (key); Kostjukov 1984: 3435 (key).
Dzhanokmenia Kostjukov: Graham 1991: 162–163 (elevated to generic rank); Kostjukov 2014: 84 (diagnosis); Kostjukov and Kosheleva 2014: 160 (diagnosis); Kostjukov and Kosheleva 2015: 451 (diagnosis); Li et al. 2016: 448 (diagnosis, key).

##### Diagnosis.

Antenna usually yellow, with 1–3 anelli, 3 funicle segments, and 3-segmented clava. Mesosoma convex, pronotum very short, transverse; midlobe of mesoscutum about as long as broad, with a single row of adnotaular setae on each side; scutellum with two distinct longitudinal submedian lines and two setae behind the middle; propodeum with median carina, without plica. Tegula yellow. Fore wing with marginal and stigmal veins very thick; submarginal vein with only one dorsal seta; marginal vein very short, much shorter than costal cell; apical margin without setae. Metasomal terga with yellow areas or completely dark.

##### Distribution.

Palearctic region: China (Xinjiang), Kazakhstan, Russia, and Turkmenistan.

##### Host.

*Stefaniola* sp. (Diptera: Cecidomyiidae); first host record for the genus.

### Key to species of *Dzhanokmenia* (females)

**Table:** 

1	Metasomal terga green, with bluish or bronze tint and metallic shine, without yellow or brownish yellow areas	**2**
–	Metasomal terga at least partially yellow or brownish yellow	**7**
2(1)	Meso- and metafemora yellow	**3**
–	Meso- and metafemora black or dark brown	**6**
3(2)	Funicular segments quadrate, as long as wide	***D. antonovae* (Kostjukov)**
–	Funicular segments longer than wide	**4**
4(3)	Funicular segments unequal in length	***D. nikolskajae* (Kostjukov)**
–	Funicular segments equal in length	**5**
5(4)	Fore wing with STV less than 0.2× as long as MV and PSV; body green, without bronze tint; apical 1/4 of procoxa and apical 2/3 of mesocoxa yellow	***D. zadepskyi* (Kostjukov)**
–	Fore wing with STV 0.3× as long as MV and PSV; body green with yellow or orange reflections; coxae green with metallic tinge	***D. karamayica* Li, Wang & Zhu**
6(2)	Funicle yellow, F2 2× as long as greatest width	***D. demakovi* (Kostjukov)**
–	Funicle brown, F2 more than 2× as long as its greatest width	***D. kurdjumovi* (Kostjukov)**
7(1)	Metasomal terga yellow; POL 9.0× OOL	***D. kozlovi* (Kostjukov)**
–	Metasomal terga only partially yellow or brownish yellow; POL at most 5.0× OOL	**8**
8(7)	Metasoma predominantly yellow with terga IV–VII brownish yellow	**9**
–	Metasoma with dark or green metallic patterns on terga	**10**
9(8)	Metasomal terga IV–VII brownish yellow; hind wing at least 9.0× as long as broad; mesoscutum with median line weakly expressed; metasoma as long as mesosoma	***D. bibikovae* (Dzhanokmen)**
–	Only metasomal tergum VI brownish yellow; hind wing at most 7.5× as long as broad; mesoscutum with median line distinct; metasoma 1.4× as long as mesosoma	***D. evgenyi* Kostjukov & Kosheleva**
10(8)	POL at most 1.6× OOL; mid lobe of mesoscutum with strong median line	**11**
–	POL at least 3.0× OOL; mid lobe of mesoscutum without median line	**12**
11(10)	Scutellum at least 1.4× as broad as long; MV at least 2.9× as long as STV; body length 1.2–1.5 mm; MV 3.7–3.75× as long as STV	***D. kasparyani* Kostjukov & Kosheleva**
–	Scutellum at most 1.25× as broad as long; MV at most 2.5× as long as STV; body length 1.9–2.0 mm; MV 3.4–3.6× as long as STV	***D. sugonjaevi* Kostjukov**
12(10)	Metasomal terga II–IV entirely yellow; metasoma longer than mesosoma (1.2×)	**13**
–	Metasomal terga II–IV not entirely yellow; metasoma shorter than mesosoma (less than 1.0×)	**14**
13(12)	Pro- and mesocoxae mostly yellow; metasoma with tergum VII with anterior triangular- to crescent-shaped area dark brown with green metallic tinge, laterally with yellow part occupying most of tergum VII; POL at least 4.5× OOL	***D. yuxuannis* Li, Wang & Hu, sp. nov.**
–	Pro- and mesocoxae mostly dark green with metallic tinge as on mesosoma; metasoma with tergum VII fully dark green with metallic tinge; POL at most 4.0× OOL	***D. stefaniolae* Li, Wang & Hu, sp. nov.**
14(12)	Vertex with a yellow area surrounding ocelli; metasomal terga I–IV laterally with round greenish-tinged spots, terga V–VI green with metallic tinge, and tergum VII yellow	***D. muleica* Li, Wang & Hu**
–	Vertex without a yellow area surrounding ocelli; metasomal terga I–IV yellow, without round greenish-tinged spots, terga V–VII green with strong metallic tinge	***D. gobica* Li, Wang & Zhu**

### Species treatment

#### Dzhanokmenia stefaniolae

Taxon classificationAnimaliaHymenopteraEulophidae

Li, Wang & Hu sp. nov.

CD71B6B7-5998-57D2-922B-185446F5388E

http://zoobank.org/9E38CB14-AF1C-4915-A0C7-749956F29057

Figs 1–6 , 7–12

##### Description.

**Figures 1–6.**
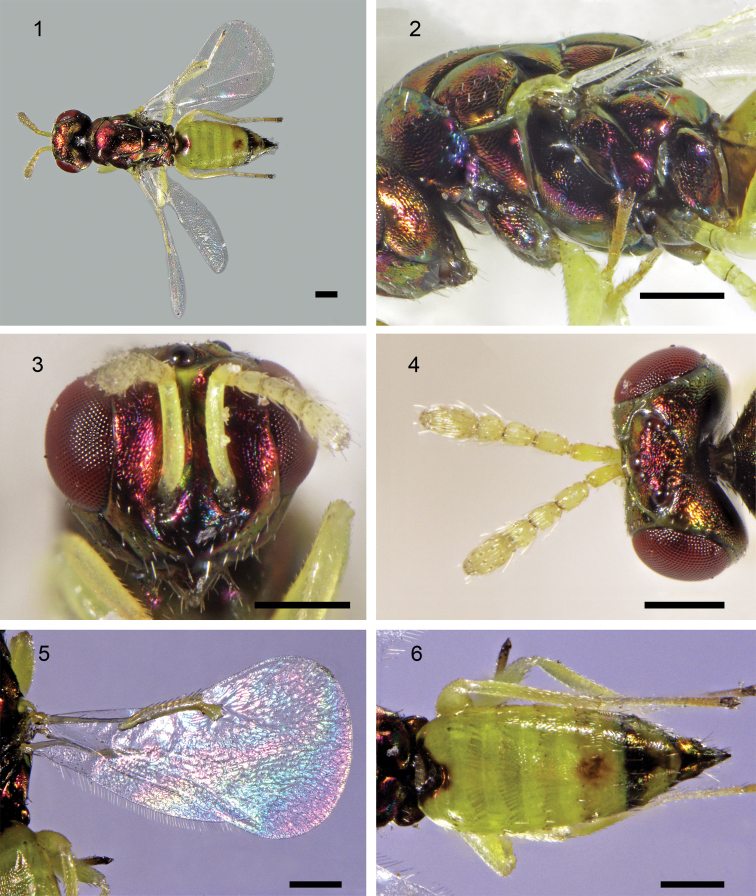
*Dzhanokmenia stefaniolae*, female (holotype): **1** habitus, dorsal **2** mesosoma, lateral **3** head, frontal **4** head, dorsal **5** wings **6** metasoma, dorsal. Scale bars: 0.2 mm.

**Female** (holotype, Figs 1–6). Body length 2.1 mm; Head with metallic tinge, vertex with small yellow area outside of each posterior ocellus, and with a triangular yellow T-shaped area in front of anterior ocellus; eye red; ocelli dark red; antenna with scape, pedicel and flagellum yellow; mandible dark brown. Mesosoma green with strong metallic tinge. Legs yellow except all coxae mostly dark green with metallic luster, as on mesosoma, and apical tarsomeres brown (Fig. 2). Metasoma with tergum I yellow except for metallic tinge in a triangular anteromedial area; terga II–IV entirely yellow; tergum V medially with a round green or orange metallic pattern; tergum VI mostly with green metallic tinge, laterally with small yellow part round like, yellow part not large than metallic tinge area; tergum VII fully dark green with metallic tinge. Ovipositor black with green metallic tinge; hypopygium fully black with green metallic tinge; outer plate of ovipositor mostly black.

***Head*** (Fig. 3) 1.1× as wide as high, 1.5× as wide as midlobe of mesoscutum. Ocellar triangle surrounded by shallow grooves (vertex collapses along these lines in air-dried specimens). POL 3.8× OOL. Torulus at lower margin of eye. Malar space about 0.4× eye height. Malar sulcus strongly curved.

***Antenna*** (Fig. 4) with scape 4.0× as long as wide, not reaching anterior ocellus; pedicel 1.6×, F1 1.6×, F2 1.5×, F3 1.0×, and clava 1.9× as long as wide; C3 short with rounded apex, without a terminal spine.

***Mesosoma*** 1.7× as long as wide, convex. Pronotum in dorsal view medially very short. Mesoscutum with midlobe about 1.2× as long as wide, with median line very weak, and with a single row of 3 or 4 adnotaular setae. Scutellum 1.2× as long as wide, with anterior pair of setae in posterior half, and submedian and sublateral grooves strong and parallel to each other. Propodeum medially as long as dorsellum, with a median carina but without paraspiracular carinae; callus with 4 or 5 setae; with a groove extending from spiracle to posterior margin of propodeum.

***Fore wing*** (Fig. 5) hyaline, 1.9× as long as wide. Speculum large, extending from parastigma to stigmal vein. Discal setae short. CC 1.6× as long as MV; MV thick, 6.0× as long as wide; MV 2.4× as long as STV, with 7 marginal setae (about 0.5× as long as STV); STV rather thick; PSV not thicker than MV, short, stub-like. Hind wing rounded apically, 4.5× as long as wide.

***Legs***. Metafemur 3.5× as long as wide.

***Metasoma*** (Fig. 6) 1.2× as long as mesosoma, 2.0× as long as wide; hypopygium extending to about half length of metasoma; cercal setae subequal in length; ovipositor sheath slightly protruding.

**Figures 7–12.**
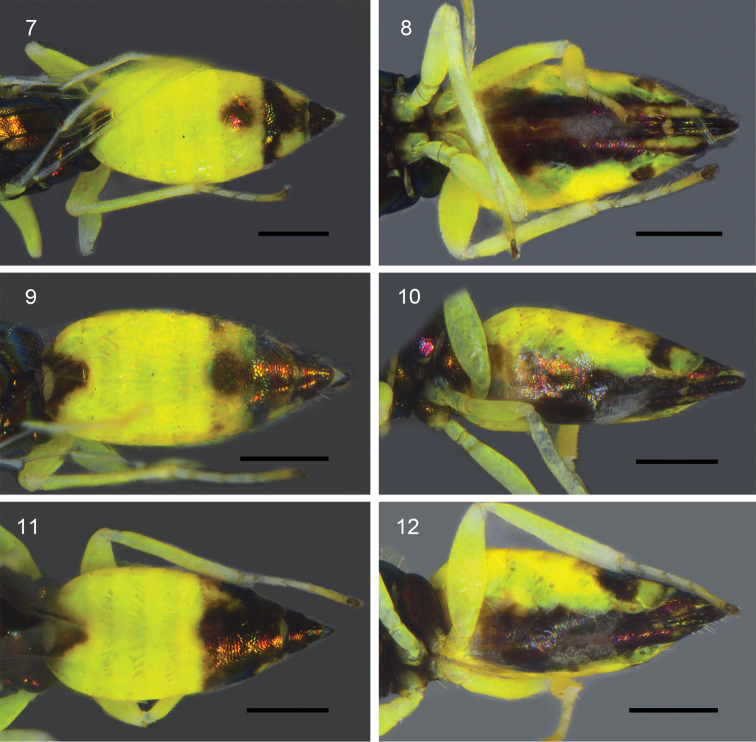
*Dzhanokmenia stefaniolae*, female (paratypes): **7, 9, 11** metasoma, dorsal **8, 10, 12** metasoma, ventral. Scale bars: 0.2 mm.

Variation (paratypes, Figs 7–12). Body length 1.9–2.1 mm. Metasomal tergum V with metallic area small to large size which occupying most of the tergum (Figs 7, 9, 11); tergum VI with metallic pattern area large to very large, relatively, with yellow part laterally from middle size to small (Figs 8, 10, 12); hypopygium from dark brown to black; outer plate of ovipositor mostly black to entirely black with metallic reflections (Figs 8, 10, 12). Pedicel 1.6–1.7×, F1 1.6–1.7×, F2 1.5–1.6×, F3 0.9–1.0×, clava 1.8–1.9× as long as wide. POL 3.8–4.0× OOL. EL 1.9–2.4× MSP. ML 1.6–1.7× MW. STL 1.1–1.2× STW. FWL 1.8–1.9× FWW. HWL 4.4–4.9× HWW. CC 1.5–1.6× MV. MV 2.3–2.4× STV. GL 2.0–2.3× GW. MFL 3.5–3.8× MFW.

**Male.** Unknown.

##### Etymology.

The species is named after the host genus.

##### Type material.

***Holotype*** f# [ICXU], air-dried on card point: China, Xinjiang, Fukang, 44°22'29"N, 87°52'57"E, 466 m, reared from stem-gall of *Stefaniola* sp. on *H. ammodendron* 26.iv.2015, H.-y. Hu group. ***Paratypes***: 3 f# on card points [ICXU], same label data as holotype.

##### Host.

**Figures 13–18.**
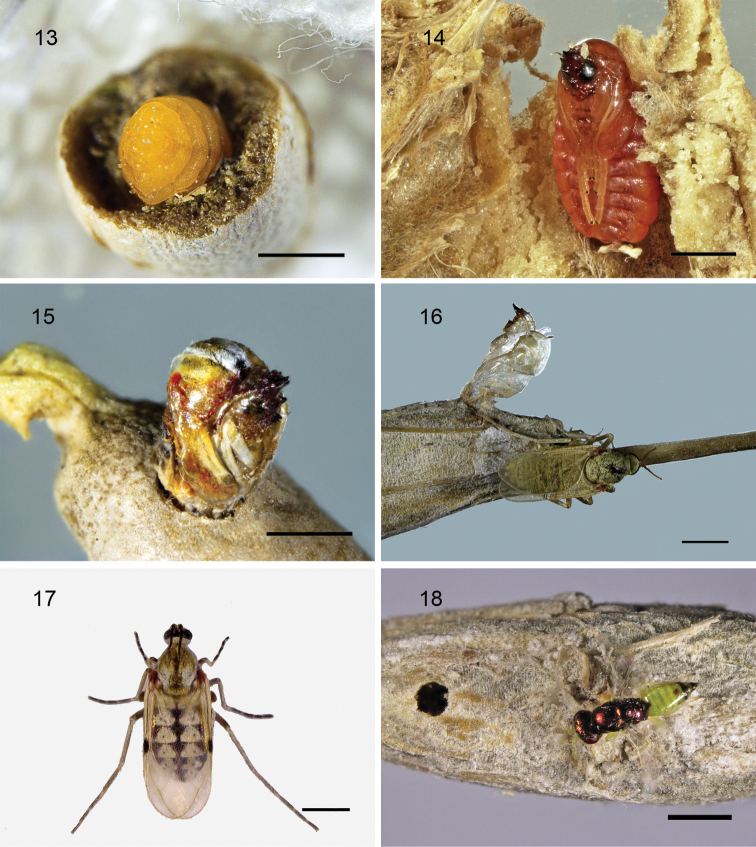
**13–17***Stefaniola* sp.: **13** larva **14, 15** pupa **16, 17** adult **18** emergence hole of adult *Dzhanokmenia stefaniolae*. Scale bars: 1 mm.

An unidentified species of *Stefaniola* Kieffer (Cecidomyiidae). The larval, pupal and adult stages of *Stefaniola* sp., are shown in Figs 13–17. The emergence hole of adult *D. stefaniolae* is shown in Fig. 18.

##### Distribution.

China: Xinjiang.

##### Remarks.

See the remarks of the other species below.

#### Dzhanokmenia yuxuannis

Taxon classificationAnimaliaHymenopteraEulophidae

Li, Wang & Hu sp. nov.

7BE4DB65-5C51-523C-B6EA-DB3FCB35F7E7

http://zoobank.org/300330F0-A903-40A4-AD84-08240A282CB2

Figs 19–26

##### Description.

**Figures 19–26.**
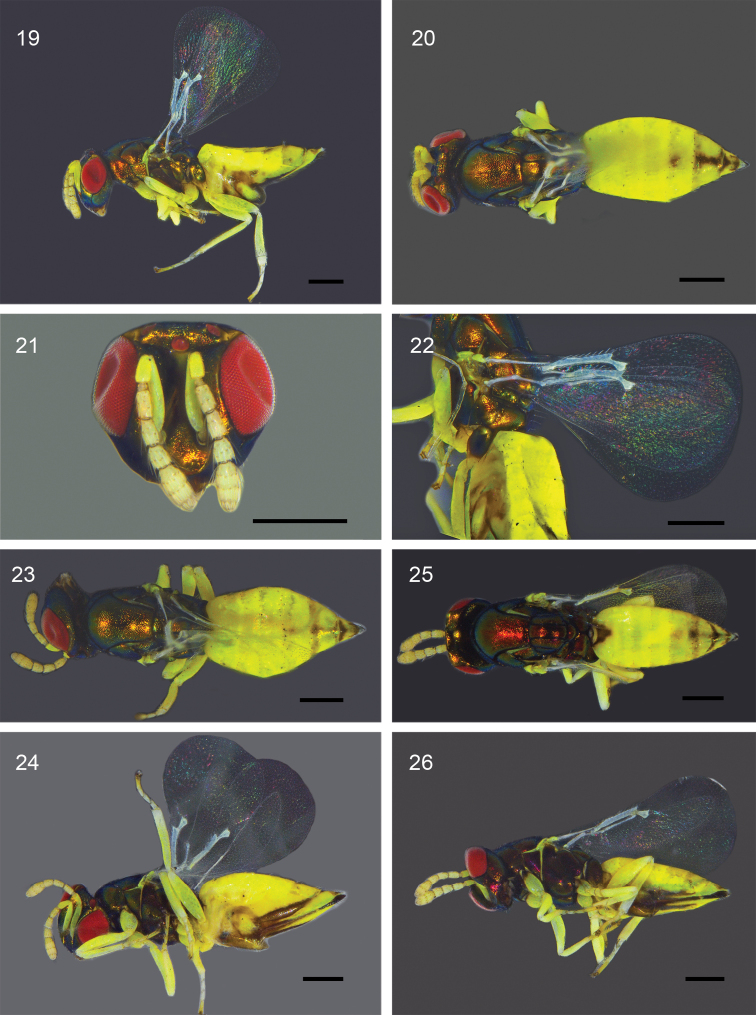
*Dzhanokmenia yuxuannis*, female (**19–22** holotype **23–26** paratypes): **19** habitus, lateral **20** body, dorsal **21** head, frontal **22** wings **23, 25** metasoma, dorsal **24, 26** metasoma, ventral. Scale bars: 0.2 mm.

**Female** (holotype, Figs 19–22). Body length 1.9 mm. Head with metallic tinge, eye red; ocelli dark red; antenna with scape, pedicel and flagellum yellow; mandible dark brown. Mesosoma with strong metallic tinge. Legs yellow except metacoxa mostly with metallic luster, as on mesosoma, and apical tarsomeres dark. Tergum I yellow except for metallic tinge in a triangular anteromedial area; terga II–IV entirely yellow; tergum V mostly yellow except medially with a faint pale brown round with faint metallic tinge; tergum VI mostly yellow, with brown stripe from anterior to posterior margins with green metallic tinge, with anterior margin brown; tergum VII with anterior triangular-shaped area dark brown with green metallic tinge, with yellow part laterally which is larger than the metallic area (Fig. 20). Ovipositor dark brown with metallic tinge; hypopygium laterally with a yellow oval area surrounded by brown to black part; upper outer plate of ovipositor with two dark brown stripe areas with metallic reflections on either side of ovipositor, and lower outer plate of ovipositor with yellow stripe-like part between dark brown stripe and ovipositor, which is linked with tergum VII.

***Head*** (Fig. 21) 1.2× as wide as high, 1.6× as wide as midlobe of mesoscutum.

Ocellar triangle surrounded by shallow grooves. POL 5.0× OOL. Malar space about 0.4× eye height. Torulus at lower margin of eye. Malar sulcus strongly curved.

***Antenna*** (Fig. 21) with scape 4.6× as long as wide, not reaching anterior ocellus; pedicel 2.0×, F1 1.7×, F2 1.5×, F3 1.1×, and clava 2.1× as long as wide; C3 short with rounded apex, without a terminal spine.

***Mesosoma*** 1.8× as long as wide, convex. Pronotum in dorsal view medially very short. Mesoscutum with midlobe about 1.1× as long as wide, with median line very weak, and with a single row of 3 or 4 adnotaular setae. Scutellum 1.15× as long as wide, with anterior pair of setae in its posterior half, submedian and sublateral grooves strong and parallel to each other. Propodeum medially as long as dorsellum, with a median carina but without paraspiracular carinae; callus with 4 or 5 setae; with a groove extending from spiracle to posterior margin of propodeum.

***Fore wing*** (Fig. 22) hyaline, 1.8× as long as wide; speculum large, extending from parastigma to stigmal vein. Discal setae short. CC 2.0× as long as MV; MV thick, 5.0× as long as wide; MV 2.2× as long as STV, with 7 marginal setae (about 0.5× as long as STV); STV rather thick; PSV not thicker than MV, short, stub-like. Hind wing rounded apically, 4.7× as long as wide.

***Legs***. Metafemur 3.3× as long as wide.

***Metasoma*** 1.2× as long as mesosoma, 2.0× as long as wide; hypopygium extending to about half length of metasoma; cercal setae subequal in length; ovipositor sheath slightly protruding.

Variation (paratypes, Figs 23–26). Body length 1.9–2.0 mm. Metasomal tergum V with pale brown round from nearly invisible to visible (Figs 20, 23, 25); tergum VI with brown stripe small to large, anterior margin pale brown to brown; tergum VII with anterior triangular-shaped to crescent-shaped area dark brown with green metallic tinge, laterally with yellow part occupying mostly area of tergum VII. Ovipositor brown to dark brown with faint green or orange metallic tinge; hypopygium with oval shaped yellow area middle size to large, which is surrounded by brown to black part with metallic tinge; upper outer plate of ovipositor with dark brown stripes narrow to wide, relatively, lower outer plate of ovipositor with yellow stripe-like part from wide to narrow (Figs 24, 26). HW 1.1–1.2× HL. Pedicel 1.9–2.0×, F1 1.6–1.7×, F2 1.5–1.6×, F3 1.0–1.1×, clava 2.0–2.1× as long as wide. POL 4.7–5.0× OOL. EL 2.1–2.5× MSP. ML 1.7–1.8× MW. STL 1.1–1.3× STW. FWL 1.8–1.9× FWW. HWL 4.6–5.4× HWW. CC 1.7–2.0× MV. MV 2.2–2.6× STV. GL 2.0–2.2× GW. MFL 3.3–3.5× MFW.

**Male.** Unknown.

##### Etymology.

The species name is a noun in apposition; it is derived from the first and second authors’ son’s first name, Yuxuan (Wang Yuxuan).

##### Type material.

***Holotype*** f# [ICXU], air-dried on card point: China, Xinjiang, Fukang, 44°22'29"N, 87°52'57"E, 466 m, sweeping on *H. ammodendron* and *H. persicum*, 26.iv.2015, H.-y. Hu group. ***Paratypes***: 2 f# on card points [ICXU], same label data as the holotype.

##### Hosts.

Unknown.

##### Distribution.

China: Xinjiang.

##### Remarks.

This species is similar to *D. stefaniolae*, distinguished from each other by the combination of features as shown in Table 1.

**Table 1.** Summary of morphological differences between *Dzhanokmenia stefaniolae* and *D. yuxuannis*.

Species/Characters	*D. stefaniolae*	*D. yuxuannis*
POL : OOL	3.8–4.0	4.6–5.0
Pedicel length to width	1.6–1.7	1.9–2.0
CC : MV	1.5–1.6	1.7–2.0
Coxae	All coxae mostly dark green with metallic luster, as on mesosoma.	Pro- and mesocoxae mostly yellow, metacoxa with metallic luster as on mesosoma.
Tergum V	Metasomal tergum V medially with a round green or orange metallic pattern from small to large size which occupying most of the tergum.	Metasomal tergum V mostly yellow except medially with a pale brown round from nearly invisible to visible with faint metallic tinge.
Tergum VI	Tergum VI mostly with green metallic tinge from large to very large, relatively, laterally with small yellow part round like, from middle size to small.	Tergum VI mostly yellow, with brown stripe from anterior to posterior margins with green metallic tinge small to large, with anterior margin pale brown to brown.
Tergum VII	Tergum VII fully dark green with metallic tinge.	Tergum VII with anterior triangular- to crescent-shaped area dark brown with green metallic tinge, laterally with yellow part occupying most of tergum VII.
Ovipositor	Ovipositor black with green metallic tinge.	Ovipositor brown to dark brown with faint green or orange metallic tinge.
Hypopygium	Hypopygium dark brown to black.	Hypopygium with oval shaped yellow area medium-sized to large surrounded by brown to black part with metallic tinge.
Outer plate of ovipositor	Outer plate of ovipositor mostly black to entirely black with metallic reflections.	Upper outer plate of ovipositor with narrow to wide dark brown stripes, lower outer plate of ovipositor with wide to narrow yellow stripe-like part.

## Discussion

The description and illustration of the two new species from the Beishawo desert and the key to the species will contribute to our understanding of *Dzhanokmenia*. The 13 described species of *Dzhanokmenia*, show quite different colour patterns of coxae, metasomal terga and hypopygium according to Dzhanokmen (1971), Kostjukov (1977, 1978, 1984, 2014), Kostjukov and Kosheleva (2014, 2015) and Li et al. (2016). We regard the differences as interspecific characters which allow the separation of species in *Dzhanokmenia* as shown in the key.

Including two new species in this paper, 15 valid species of *Dzhanokmenia* are known from the arid to semi-arid regions of Southern Russia and Central Asia. However, hosts remain to be discovered for any of the species. Li et al. (2016) suggested that they are most likely associated with *Haloxylon* and *Tamarix* spp., feeding on them or as parasitoids of their pests. In this study, *D. stefaniolae* was reared from ball-like stem-galls of *Stefaniola* sp. on *H. ammodendron* for the first time. *D. yuxuannis* was collected by sweeping from both *H. ammodendron* and *H. persicum* at the same collecting site. This suggests a relationship between *D. yuxuannis* and *Haloxylon* spp., but it needs further study.

Besides *D. stefaniolae*, from ball-like stem-galls of *Stefaniola* sp. on both *H. ammodendron* and *H. persicum* in the same desert localities, we also reared *Mesopolobus quadrimaculatus* Dzhanokmen (Pteromalidae), *Aprostocetus* sp. (Eulophidae), *Psyllaephagus caillardiae* Sugonjaev (Encyrtidae), and also some unidentified Eurytomidae and Platygastridae (Hymenoptera) (specimens in ICXU). The relationships among these species and *Stefaniola* sp. are not clear and need further study. Currently, the taxonomy of the Palaearctic species of *Stefaniola* is in flux, so unfortunately this *Stefaniola* sp. cannot be positively identified to species (pers. comm. by Ke-Long Jiao).

## Supplementary Material

XML Treatment for Dzhanokmenia

XML Treatment for Dzhanokmenia stefaniolae

XML Treatment for Dzhanokmenia yuxuannis
